# Epithelial-Mesenchymal Transition in Oral Squamous Cell Carcinoma

**DOI:** 10.5402/2012/681469

**Published:** 2012-03-29

**Authors:** Suttichai Krisanaprakornkit, Anak Iamaroon

**Affiliations:** Department of Oral Biology and Diagnostic Sciences, Faculty of Dentistry, Chiang Mai University, Chiang Mai 50200, Thailand

## Abstract

Oral cancer is one of the drastic human cancers due to its aggressiveness and high mortality rate. Of all oral cancers, squamous cell carcinoma is the most common accounting for more than 90%. Epithelial-mesenchymal transition (EMT) is suggested to play an important role during cancer invasion and metastasis. Recently, emerging knowledge on EMT in carcinogenesis is explosive, tempting us to analyze previous studies on EMT in oral squamous cell carcinoma (OSCC). In this paper, we have first addressed the general molecular mechanisms of EMT, evidenced by alterations of cell morphology during EMT, the presence of cadherin switching, turning on and turning off of many specific genes, the activation of various signaling pathways, and so on. The remaining part of this paper will focus on recent findings of the investigations of EMT on OSCC. These include the evidence of EMT taking place in OSCC and the signaling pathways employed by OSCC cells during their invasion and metastasis. Collectively, with the large body of new knowledge on EMT in OSCC elaborated here, we are hopeful that targeting treatment for OSCC will be developed.

## 1. Introduction

Epithelial-mesenchymal transition (EMT) is considered a reversibly biologic process that is important for normal embryogenesis and organ development from single-layered to multilayered organisms, particularly during gastrulation and neural crest cell migration [1]. The neural crest cells, originated from nonneural ectodermal cells that are situated above the neural tube, can migrate from the ectodermal layer through the disrupted basal lamina by acquiring the complex process of EMT. In this process, the neural crest cells will ultimately change their epithelial phenotype to the fibroblast-like morphology and eventually migrate to a distant site to form new organs. Once reaching their destinations, where they become differentiated into different cell types, they are likely to undergo the reverse process, called mesenchymal-epithelial transition (MET) that involves the conversion of mesenchymal back to epithelial cells. Therefore, the epithelial cells appear to be plastic and are able to change back and forth between EMT and MET. One of the best-studied and -known processes of EMT is the breakdown of intercellular adhesion between epithelial cells by inhibiting expression of E-cadherin (epithelial cadherin), whereas the cells that undergo the EMT process aberrantly express higher levels of N-cadherin (mesenchymal cadherin). This process is called “cadherin switching” [2, 3], which will be discussed later in this paper.

In addition to the importance of EMT process during the prenatal development, EMT is a natural process that can be found in several pathophysiological conditions in human adults. Particularly, EMT is a necessary component of the inflammatory process and normal wound healing in adulthood [4]. However, dysregulated EMT in the inflammatory process and wound healing can pathologically result in scar formation and then lead to fibrotic lesions in human organs, especially in kidney [5]. By analogy to the EMT process undertaken by the neural crest cells, the delamination of malignant cells that undergo metastasis and invasion into adjacent underlying tissue from the primary tumor can also exploit this process. Accordingly, it has been demonstrated by a great number of studies that the process of EMT is linked with cancer cell metastasis and invasion, which directly correlates with poor prognostic markers. These include poor tumor staging, an increased risk of cancer recurrence, and a decreased survival rate in several types of cancers, such as, breast cancer [6], colorectal cancer [7, 8], gastric cancer [9], bladder cancer [10], and lung cancer [11, 12]. Nevertheless, the transient nature and differing degrees of cells undergoing EMT events that occur only in some cancer cells at the tumor front, while other cancer cells still retain their epithelial traits, make scientific investigation of this phenomenon *in vivo* difficult. Therefore, much of what has been learned about the EMT is derived from *in vitro* studies that have been correlated with clinical observations in tumor specimens. 

The phenomenon of EMT can be simply divided into 3 distinct types [13], including the type 1 EMT in embryogenesis, the type 2 EMT in the wound healing, tissue regeneration, and organ fibrosis, and the type 3 EMT in cancer cell metastasis and invasion. In the type 3 EMT, several markers of EMT in cancer specimens have been associated with the presence of metastasis, the increased recurrence rate, and the decreased survival rate, supporting the concept that several events taking place in the type 3 EMT represent aggressive behavioral changes of cancer cells. As more knowledge accumulated from studies in the EMT area, we will have a better insight into the similarities and differences among these three types of EMT in the future. In this paper, an overview of our current understandings of the type 3 EMT at the molecular level will be delineated, including loss of cell to cell adhesion by activation of transcriptional repressors, actin reorganization and formation of invadopodia, expression of relevant matrix metalloproteinases (MMPs), and changes in the expression of microRNAs. In addition, some evidence derived from our study in oral squamous cell carcinoma (OSCC) tissues and cell lines will be demonstrated to support the EMT concept for the aggressiveness of OSCC. It is recommended that the readers consult other reviews of the literature for more details of the type 1 and type 2 EMT [13–15].

## 2. Loss of Cell-to-Cell Adhesion by Transcriptional Repressors and Its Consequences

 The loss of cell to cell adhesion, triggered by activation of an EMT program, together with subsequent cell-ECM adhesions mediated by integrins, like *β*4, *α*5*β*1, and *α*V*β*6 [16], is among the first critical steps of cancer metastasis, invasion, and progression. Several reports have shown the acquisition of mesenchymal markers in carcinoma cells, such as vimentin, N-cadherin, and fibronectin, with the concomitant loss of epithelial E-cadherin, a major constituent of the adherens junctions, in the processes of EMT. Consequently, E-cadherin is considered to be a “master regulator” of EMT. Furthermore, the connection between loss of E-cadherin expression in cancer cells and poor patient prognosis, including increased tumor grade, metastasis, and mortality, has been established by several studies. There are a number of causative factors that can result in functional loss of E-cadherin during tumor progression in epithelial carcinoma. These include mutations of E-cadherin in germ-line and somatic cells, chromosomal aberrations, DNA hypermethylation of the E-cadherin gene, transcriptional suppression of E-cadherin by several transcriptional repressors whose activities are induced by growth factors, and the cleavage of E-cadherin by proteinases [17]. It is interesting to note that the existence of genetic mutations of E-cadherin is extremely rare in almost every type of cancer, whereas the epigenetic modification by DNA hypermethylation of the promoter region of E-cadherin gene is more frequently found and responsible for the EMT process in cancer cells [17].

**Figure 1:**
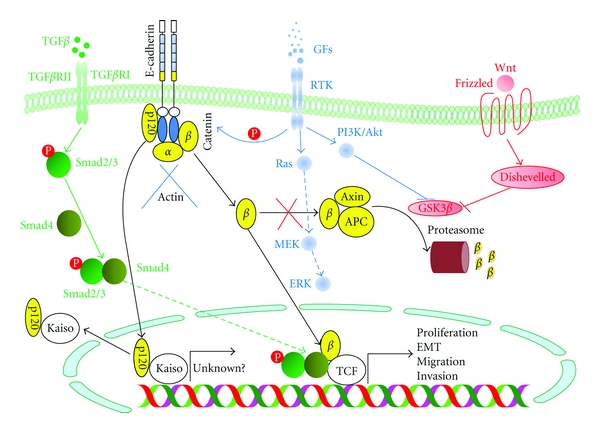
The pivotal role of E-cadherin, *β*-catenin, and transforming growth factor *β* (TGF*β*) and Wnt signaling pathways in an epithelial-mesenchymal transition (EMT) program. Upon loss of E-cadherin expression, *β*-catenin, which colocalizes with E-cadherin at the cytoplasmic membrane, is released and accumulated in the cytoplasm. Under normal circumstances, liberated *β*-catenin is rapidly degraded by proteasomes in the adenomatous polyposis coli (APC)/axin/glycogen-synthase kinase 3*β* (GSK3*β*) complex. However, as in several cases of cancers, APC is not functional or the activity of GSK3*β* is diminished by the activation of Wnt signaling pathway, *β*-catenin can migrate to the nucleus, where it functions as a cofactor for the transcription factor T-cell factor (TCF) and potentiates its transcriptional activity in concert with the phosphorylated SMAD 2 and 3, triggered by TGF*β* signaling, leading to tumor proliferation, EMT, migration, and invasion. Similar to *β*-catenin, p120-catenin can translocate into the nucleus, where it binds to the transcriptional repressor Kaiso to deactivate this protein, causing the unknown genes to become activated by derepression. GFs: growth factors; RTK: receptor tyrosine kinase; TGF*β*R: TGF*β* receptor.

 There are several growth factors, probably emanating from tumor-associated stromal cells, that induce the loss of E-cadherin expression/function and then cancer cell migration and invasion, including interleukin-6 [18], transforming growth factor *β* (TGF*β*) [19], hepatocyte growth factor and its receptor, Met [20], epidermal growth factor [21], insulin-like growth factor [22], and fibroblast growth factor (FGF) [23]. For instance, upon induction of EMT by TGF*β*, an increase in DNA methylation of the E-cadherin that prevents the transcription of E-cadherin gene has been observed, whereas withdrawal of TGF*β* promotes a reversion of EMT process by triggering the reexpression of E-cadherin [24]. Other possible signaling mechanisms of TGF*β* that are associated with EMT include the activation of Sma and mothers against decapentaplegic (Smad) proteins (Figure 1) that facilitate cell motility and autocrine production of TGF*β*, which can further enhance the EMT program [25, 26], and the activation of mitogen-activated protein kinase (MAPK) and PI3K/Akt pathways independent of Smad activation [27]. Consistently, a better prognosis of mice in the model of skin and colon cancers has been obtained from the deficiency of TGF*β* receptor expression [28, 29].

Upon binding to their putative receptors, these growth factors can activate the intracellular signaling pathways that activate several transcriptional repressors of E-cadherin gene transcription, such as Snail, Slug (now called Snail2) [30–32], zinc finger E-box binding homeobox (ZEB) 1, ZEB2, and Twist (a transcription factor known to drive EMT and metastasis) [33, 34]. Binding between these repressors and the E-cadherin gene promoter can lead to the epigenetic silencing of E-cadherin expression in a number of ways. For example, the Snail, a member in the family of zinc-finger transcription factors that is proven to be a master gene for EMT, induces the activity of histone deacetylase enzymes to remove the acetyl groups from the histone proteins, resulting in a high-affinity binding between histones and DNA, which prevents the transcription of E-cadherin gene [35]. Furthermore, the histone deacetylation can enhance the binding between polycomb repressor complex 2 and DNA, causing DNA hypermethylation that can suppress the E-cadherin gene transcription [36]. Otherwise, Snail may upregulate ZEB1 expression, which further inhibits E-cadherin expression. Consistent with the inhibitory effect of Snail on E-cadherin expression, a reverse correlation between the expression of E-cadherin and that of Snail has been demonstrated in patients suffering from OSCC [37]. As with the inhibitory effect of Snail on E-cadherin expression, forced overexpression of Slug markedly reduces E-cadherin expression, while significantly enhances expression of MMP2, resulting in tumor metastasis *in vivo* [11].

An impairment of E-cadherin function can occur from proteolytic cleavage by *γ*-secretase and a disintegrin and metalloproteinase (ADAM) 10 that generates E-cadherin fragments both inside and outside the cell [38, 39]. It has been shown that the shed extracellular domain of E-cadherin interferes with the cell to cell adhesion, enhancing cell detachment and then migration [40]. In addition, the cytoplasmic fragment of E-cadherin can translocate into the nucleus, where it modulates the activity of transcriptional repressor, Kaiso, which exerts its suppressive activity on the promoter region of target genes that are still largely unknown [41]. Besides the proteolytic cleavage of E-cadherin, degradation of E-cadherin in the adherens junctions by endocytosis, ubiquitination, and subsequent proteasomal degradation has been shown by phosphorylation of E-cadherin upon activation of receptor tyrosine kinases (Figure 1), such as growth factor receptors, and nonreceptor tyrosine kinase Src [42].

Subsequent to loss of E-cadherin function, which results in the disruption of adherens junctions and the loss of cell polarity, *β*-catenin, which colocalizes with E-cadherin to the cytoplasmic membrane, is critical for linking intercellular adherens junctions with the intracellular actin microfilament, and plays an essential role in the canonical Wnt signaling pathway, will be released and accumulated in the cytoplasm (Figure 1). Under normal circumstances, liberated *β*-catenin is quickly phosphorylated by glycogen-synthase kinase 3*β* (GSK3*β*) in the adenomatous polyposis coli (APC)/axin/GSK3*β* complex and subsequently destroyed by ubiquitination at the proteasomes (Figure 1) [43]. However, as commonly found in several cases of cancers, whereby the tumor suppressor APC is not functional or the activity of GSK3*β* is diminished by the activation of Wnt signaling pathway, an excessive amount of *β*-catenin in the cytoplasm can migrate to the nucleus, where it functions as a cofactor for the transcription factor T-cell factor (TCF) and potentiates its transcriptional activity in concert with the activated SMAD 2 and 3, triggered by TGF*β* signaling (Figure 1) [44]. This results in upregulated expression of a number of target genes required for tumor proliferation, migration, and invasion, such as c-Myc, cyclin D1 and D2, Slug, matrilysin, vimentin, and fibronectin [45–48]. Accordingly, shuttling of *β*-catenin from the cytoplasmic membrane to the nucleus has been detected in several different solid tumors and cancer cell lines, indicating that *β*-catenin plays a pivotal role in EMT regulation.

In addition to *β*-catenin, p120-catenin, which is also located in the E-cadherin complex, can be released and accumulated in the cytoplasm upon the dissociation of this complex (Figure 1). It has been demonstrated that p120-catenin can down-regulate the enzymatic activity of RhoA, but instead upregulate the activity of Rac and Cdc42 [49, 50]. These three GTPase enzymes function in regulating actin assembly and play an important role in the stability of cell-cell adhesion by RhoA, and the cell migration and the formation of migratory membrane protrusions, like lamellipodia by Rac and filopodia by Cdc42. Consequently, liberated p120-catenin can cause the instability of cell-cell adhesion and induce cell migration through the formation of lamellipodia and filopodia. Moreover, similar to *β*-catenin, p120-catenin can translocate into the nucleus, where it binds to the transcriptional repressor Kaiso to deactivate this protein, causing the target genes to become activated by derepression (Figure 1) [51]. However, the target genes for the p120/Kaiso complex have not yet been defined.

## 3. Formation of Invadopodia

 Lamellipodia and filopodia are alternatively defined as invadopodia, whose role is related to ECM degradation and cell migration, since they contain MMPs that are responsible for ECM degradation as well as regulation of extracellular signaling cascades [52]. It is interesting to note the formation of invadopodia in cancer cells of head and neck squamous cell carcinoma that undergo EMT [53]. Among the MMPs, membrane type 1-matrix metalloproteinase (MT1-MMP or MMP-14) plays an essential role in cell invasion and migration [54]. MT1-MMP expression was originally reported in lung carcinoma and stromal fibroblasts near the advancing tumor front [55]. Subsequently, similar patterns of MT1-MMP expression have also been described in several other carcinomas, such as colon, breast, bladder, head and neck, and cervical cancers. One of the main functions of MT1-MMP is to cleave and activate the latent form of MMP-2 into the active form. Correspondingly, the presence of both MT1-MMP and the active form of MMP-2 often correlates with poor patient prognosis [54].

## 4. Cadherin Switching

 The cadherin switching from E-cadherin to N-cadherin expression during EMT appears to provoke cell migration and invasion and correlates with poor prognosis [56, 57]. It has been demonstrated that the transcriptional repressor Twist is involved with the induction of N-cadherin in addition to its suppressive effect on E-cadherin expression [58, 59]. The aggressive behaviors of cancer cells during the cadherin switching may be explained by the findings that show the interactions between N-cadherin and platelet-derived growth factor receptor (PDGFR) [60] and between N-cadherin and fibroblast growth factor receptor (FGFR). The interaction between N-cadherin and PDGFR is known to induce several cellular events important for EMT, including actin reorganization, proliferation, and migration [61], whereas the interaction between N-cadherin and FGFR can prevent the internalization of FGFR upon binding to FGF, resulting in a prolonged MAPK activation as well as increased mobility, MMP secretion, and invasiveness of cancer cells [62]. Moreover, N-cadherin itself can upregulate the expression of FGFR, resulting in cellular resistance to apoptosis among metastasized cells that dissociate from the epithelium [63].

 As with the proteolytic cleavage of E-cadherin, the proteolytic processing of N-cadherin by ADAM10 and *γ*-secretase can generate a shed extracellular domain and an intracellular fragment, potentially possessing a signaling function as well. It has been revealed that the cytoplasmic fragment of N-cadherin can bind to transcription factor cyclic AMP-responsive element binding protein and induce its degradation at the proteasomes [64]. Nevertheless, the actual consequence of this repression by the cytoplasmic fragment of N-cadherin to tumor metastasis and invasion is still unknown.

## 5. Significance of MicroRNAs in EMT

Expression of different types of noncoding microRNAs has been reported to act both as a facilitator and as an inhibitor of the EMT program. For instance, microRNA 200 and microRNA 205 can block the suppressive activity of transcriptional repressors of E-cadherin expression, including ZEB1 and ZEB2, thus retaining the epithelial characteristics of breast cancer, whereas lack of microRNA 200 is implicated in the promotion of EMT by up-regulating vimentin expression and diminishing E-cadherin expression in breast cancer cells [65–68]. On the contrary, microRNA 21 acts in the opposite direction by facilitating TGF*β*-induced EMT [69]. Consequently, it is interesting to further investigate the role of these microRNAs in the EMT program of OSCC.

## 6. EMT in OSCC

Oral squamous cell carcinoma is one of the major leading cancers worldwide. The incidence of OSCC remains constant but appears increased in some parts of the world [70, 71]. Although OSCC is relatively easily accessible to early diagnosis and treatment, the mortality rate is still high due to the failure to control tumor recurrence and metastasis. The overall five-year survival rate for oral cancer is considerably lower than other cancers and has not significantly changed during the last two decades. Recently, novel treatments, aiming to target specific molecules, aberrantly expressed during OSCC carcinogenesis, have been investigated and tested in clinical trials at several research settings with promising results. These treatments offer hope for replacing conventional modalities considered nonspecific and caused unwanted severe complications in patients.

**Figure 2:**
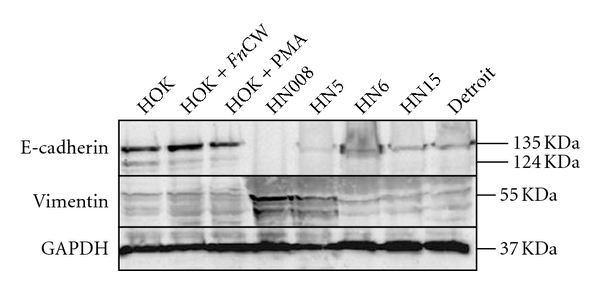
Western blot analysis of expression for vimentin and E-cadherin proteins in whole cell lysates. Up-regulation of vimentin protein was evidently demonstrated in HN008 and HN5 cell lines, while E-cadherin protein was down-regulated in all five oral cancer cell lines in comparison with a normal oral keratinocyte cell line in the presence or absence of stimulation with 10 *μ*g/mL of the cell wall extract of *Fusobacterium nucleatum* (*Fn*CW), a gram-negative periodontal bacterium, or 10 ng/mL of PMA (a phorbol ester and epithelial activator) overnight. Expression of glyceraldehyde-3-phosphate dehydrogenase (GAPDH), serving as a housekeeping gene control, was approximately equivalent among different samples, indicating equal loadings. The molecular weight of each protein was as predicted.

**Figure 3:**
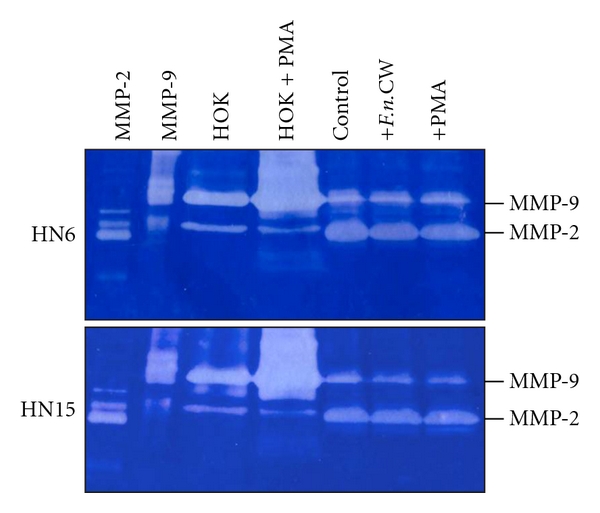
Gelatin zymography shows MMP-9 and MMP-2 activities in the culture medium of control and stimulated normal oral epithelial cells (HOK) and two oral cancer cell lines (HN6 and HN15) with 10 ng/mL of PMA or 10 *μ*g/mL of *F.n.*CW. The result showed that the phenotype of two oral cancer cell lines was indeed transformed into mesenchymal cells, in which they possessed higher MMP-2, but less MMP-9, activities than normal oral epithelial cells (HOK). Interestingly, unlike normal oral epithelial cells, there was no alteration in terms of MMP-2 and MMP-9 activities in HN6 and HN15 stimulated with either *F.n.*CW or PMA when compared to normal cells stimulated with PMA (HOK + PMA). The gelatinolytic activities of purified MMP-2 and MMP-9 are shown on the two left columns.

**Figure 4:**
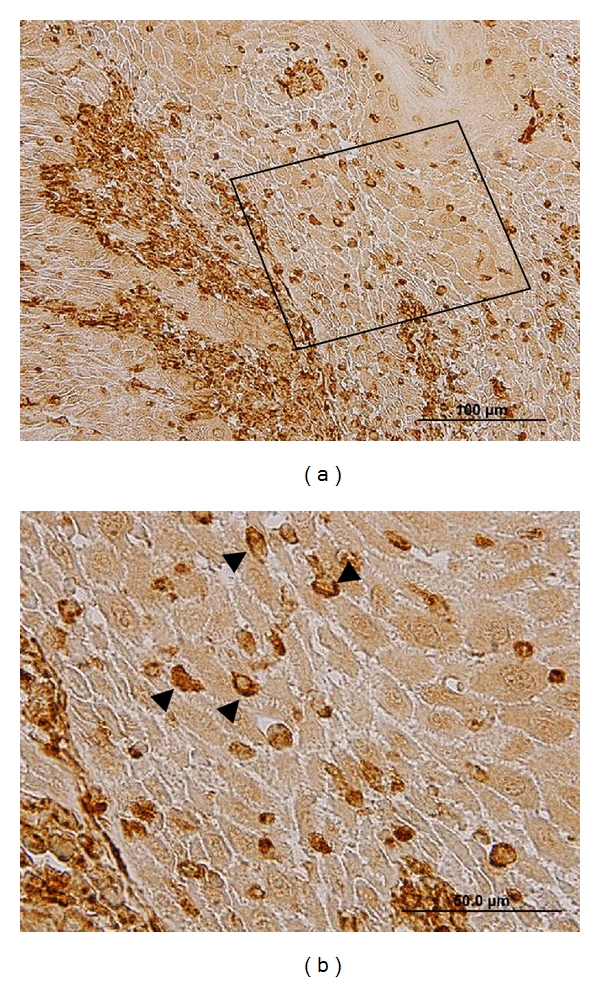
Immunohistochemical study on OSCC tissue showing that some epithelial cells at the invasive tumor fronts with the brown positive staining underwent EMT by increased cytoplasmic production of vimentin (arrowheads in (b)). (b) is a higher magnification view from an inset in (a).

EMT is the process by which epithelial cells adopt a mesenchymal phenotype or fibroblast-like properties. The epithelial cells undergoing EMT involve reorganizing their cytoskeleton, stretching out, and breaking connections with their neighbors. After they have completed the transition, those cells dissolve the extracellular matrix that restrains them and start spreading to the surrounding tissue. In general, cells proceeding EMT exhibit down-regulation of many epithelial markers, including E-cadherin, desmoplakin, cytokeratins, claudins, occluding, and beta-catenin, and up-regulation of mesenchymal markers, including N-cadherin, vimentin, fibronectin, and Snail-1/2 [72–74]. Morphologically, cancer cells undergoing EMT switch their epithelial characteristics (cobblestone-like, nonmotile and noninvasive) to their mesenchymal elongated, motile, and invasive characteristics [75]. Very recently, we have found down-regulation of E-cadherin and MMP-9, the epithelial phenotypes, and up-regulation of vimentin and MMP-2, the mesenchymal phenotypes, in OSCC cell lines (Figures 2 and 3). Vimentin, in particular, was immunolocalized in the cytoplasm of OSCC cells at the invasive tumor fronts (Figure 4).

Cadherin switching, the loss of E-cadherin expression and the gain of N-cadherin expression, is a crucial event of EMT in human cancers. As mentioned earlier, Snail is a master gene in regulating E-cadherin during the process of EMT. In an OSCC model, Snail-transfected cells showed complete EMT phenotypes with a fibroblast-like appearance, vimentin filaments, E-cadherin/N-cadherin switching, and lack of hemidesmosomes [76]. Additionally, ZEB-1 and ZEB-2 were upregulated in these cells. In a head and neck SCC (HNSCC) model, the immunohistochemical results revealed high expression of N-cadherin in the majority of HNSCC cases and the expression of N-cadherin significantly correlated with malignant behaviors [77]. Cadherin switching was found in 30 of 80 HNSCC cases and correlated with histologic differentiation, pattern of invasion, and lymph nodes metastasis. HNSCC cells also showed cadherin switching with EMT features. Similarly, spindle cell carcinoma (SpCC), a biphasic tumor composed of conventional SCC and a malignant spindle cell component, demonstrated cadherin switching [78]. The majority of cases of SpCC with metastasis also exhibited high expression of N-cadherin.

As mentioned earlier, the integrin has been found to play an important role in EMT. The *α*V*β*6 integrin, in particular, was postulated to modulate EMT in OSCC [79]. When the full length *β*6 integrin was expressed in poorly invasive OSCC cells, OSCC cells increased expression of vimentin and reduced expression of keratin and E-cadherin, while OSCC cells with the expression of the truncated form of *β*6 subunit retained their epithelial characteristics and did not alter vimentin or E-cadherin expression. In addition, the *β*6 integrin increased MMP-3 activation and tenascin-C expression. Notably, both molecules were MEK dependent.

## 7. What Signals EMT in OSCC?

**Figure 5:**
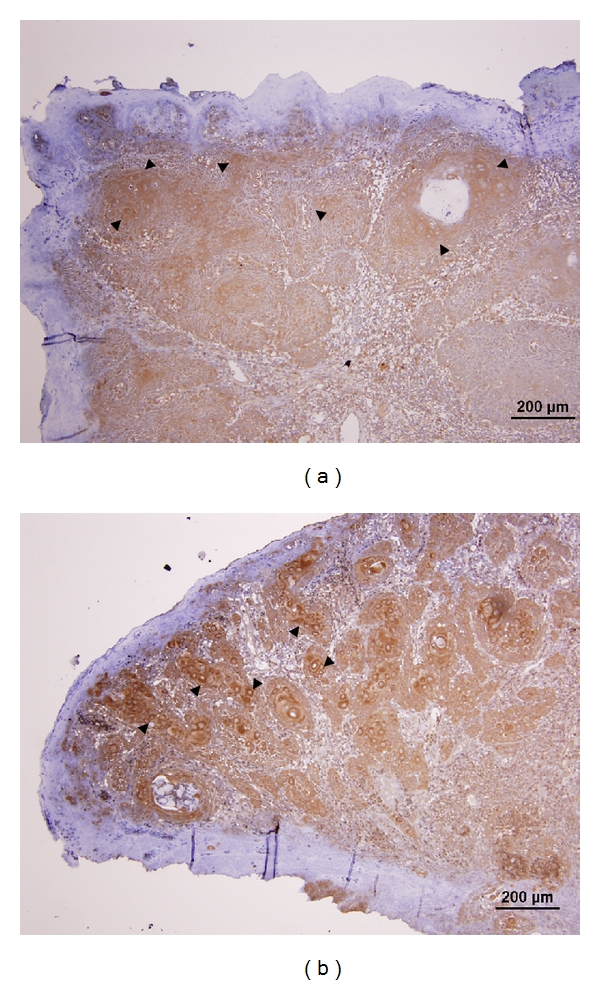
Immunohistochemical study on the expression of Akt1 and Akt2 from sections of an OSCC sample. Intense cytoplasmic staining of both Akt1 (arrowheads in (a)) and Akt2 (arrowheads in (b)) is observed in the tumor cells, while the overlying epithelium is principally negatively stained.

 Activation of the PI3K/Akt signaling pathway is a frequent event in human cancers, including OSCC (Figure 5) [80, 81]. Particularly, Akt-1 and Akt-2 were overexpressed in OSCC (Figure 5). Previous studies have shown involvement of PI3K/Akt signaling pathway in EMT of SCC. In an *in vitro* model of laryngeal SCC, epidermal growth factor (EGF) was suggested as an inducer for EMT [82]. Upon stimulation of epidermal growth factor receptor (EGFR) with EGF, SCC cells increased motility, migration, and invasion and underwent EMT with down-regulation of E-cadherin and up-regulation of N-cadherin and vimentin. Moreover, it was found that MMP-9 mediated degradation of E-cadherin into a soluble form. With the use of inhibitors of EGFR, PI3K, and MEK-1/2, EMT appeared to occur independently through two signaling pathways: PI3K/Akt and MEK-1/2/ERK-1/2 pathways.

 Similarly, an activation of the PI3K/Akt signaling pathway was shown to be an important feature of EMT in OSCC. The role of Akt in the biology of OSCC was investigated by employing OSCC lines engineered to express constitutively active Akt [74]. The results revealed that OSCC cells underwent EMT characterized by down-regulation of E-cadherin, desmoplakin, and beta-catenin and up-regulation of vimentin. Morphologically, OSCC cells lost epithelial cell characteristics and acquired fibroblast-like properties. In addition, EMT was accompanied by reduced cell-cell adhesion, increased cell motility on fibronectin-coated surfaces, and increased invasiveness in animals. Interestingly, when Akt activity was inhibited, OSCC cells acquired the mesenchymal-epithelial reverting transition and re-expressed E-cadherin; hence, the epithelial characteristics were restored [81]. This phenomenon is an important step of cancer cells to dwell in the metastatic sites and adapt to their new microenvironment. It was suggested that a strategy involving Akt inhibition be a promising therapeutic approach in controlling cancer invasion and metastasis in OSCC patients.

 TGF*β* is a paradoxical cytokine in its functional role. TGF*β* acts as a tumor suppressor in normal epithelial cells and cancer cells at their early stages of carcinogenesis. As the carcinogenesis progresses, cancer cells can switch their responses to TGF*β* and utilize TGF*β* as a potent oncogenic activator [83]. Several studies have shown that TGF*β* mediates EMT in various developmental processes and human cancers, including OSCC [84]. A study by Qiao et al. revealed that upon stimulation with recombinant TGF*β*1, Slug and MMP-9 were upregulated, while Snail expression increased and fell, in concert with the expression of MMP-2 in OSCC cells, suggesting that both Snail and Slug act as regulators of TGF*β*1-triggered EMT [85]. Interestingly, a study by Richter et al. showed that EMT in OSCC was mediated by multiple growth factors, including EGF and TGF*β*1 [86]. In contrast to the effect of treatment with either EGF or TGF*β*1, costimulation induced phenotype transition in OSCC cells with up-regulation of vimentin and down-regulation of E-cadherin. Moreover, OSCC cells displayed an enhanced invasiveness and adhesion to type I-IV collagens.

 Another study also demonstrated that the expression of MMP-10 was increased following treatment of premalignant human keratinocytes, HaCaT cells, with a combination of TGF*β*1 and EGF [87]. TGF*β*1 alone could upregulate plasminogen activator inhibitor type-1 (PAI-1) in HaCaT cells. CDK2-AP1 (p12), a downstream effector of TGF*β*, could also induce EMT of hamster cheek pouch carcinoma-1 cells by promoting the expression of Twist-2 [88]. Interestingly, those EMT cancer cells showed increased invasiveness but decreased metastatic phenotype. Collectively, TGF*β* and EGF may play a complex role in EMT as well as modulating ECM degradation and facilitating the progression of OSCC carcinogenesis.

 The Wnt/*β*-catenin pathway is one of the major signaling pathways in cell proliferation, oncogenesis, and EMT. A recent study showed that aberrant cytoplasmic accumulation of *β*-catenin induced TCF/LEF-mediated transcriptional activity, up-regulation of MMP-7, and induced EMT in OSCC cells, hence, enhancing invasion and migration of OSCC cells [89]. Recently, Iamaroon et al. found an aberration of TGF*β* signaling pathway in OSCC as evidenced by a reduction or deletion of Smad4 expression [90]. These findings suggest that some OSCC might employ an alternative signaling pathway, like the PI3K/Akt, MEK/ERK or Wnt/*β*-catenin pathway.

 Hypoxia has been found to play a critic role in EMT [91]. The hypoxic microenvironment, in particular, is common to any cancer cells and can trigger EMT via regulating the expression and activity levels of major transcriptional repressors. In tongue SCC tissues, hypoxia inducible factor (HIF)-1*α*, HIF-2*α*, and Twist-2 were overexpressed and the overexpression of these molecules, except HIF-2*α*, was associated with a shorter disease-free survival [92]. It was suggested that coexpression of more than two markers from HIF-1*α* and Twist2 be a significant prognostic predictor in patients with tongue SCC.

## 8. Conclusion

Oral squamous cell carcinoma is a devastating disease and remains a major threat to global public health. Extensive studies with impressive results have been performed and elucidated the complex nature of OSCC carcinogenesis. Emerging knowledge gained from studies on EMT in the last decade has provided a better understanding of the mechanisms of EMT in human cancers, including OSCC. Undoubtedly, this knowledge will contribute significant advances to the biology of carcinogenesis, leading to development of new biomarkers for diagnosis and prognosis and targeted therapeutics for patients suffered from OSCC.
